# Recent advances in molecular genetics of melanoma progression: implications for diagnosis and treatment

**DOI:** 10.12688/f1000research.8247.1

**Published:** 2016-06-28

**Authors:** Iwei Yeh

**Affiliations:** 1 Department of Dermatology, University of California San Francisco, San Francisco, CA, USA

**Keywords:** melanoma, dermatopathology, melanocytic neoplasia, BRAFV600E, BRAF, nevi, Spitz nevi

## Abstract

According to the multi-step carcinogenesis model of cancer, initiation results in a benign tumor and subsequent genetic alterations lead to tumor progression and the acquisition of the hallmarks of cancer. This article will review recent discoveries in our understanding of initiation and progression in melanocytic neoplasia and the impact on diagnostic dermatopathology.

## Initiating oncogenes in melanocytic neoplasia

If an initiating oncogene causes tumor formation, it should be present clonally in benign neoplasms and occur in a mutually exclusive pattern with other initiating events. BRAF ^V600E^ satisfies these criteria in melanocytic neoplasia. Studies demonstrate that *BRAF* mutations are typically present in all or none of the cells within nevi and melanomas ^1, 2^. In a recent study of the genetic evolution of melanoma, sequencing of known oncogenes in melanoma and cancer did not reveal additional driver alterations in unequivocally benign nevi with BRAF ^V600E^, additionally supporting the hypothesis that BRAF ^V600E^ mutation can initiate melanocytic nevi ^3^.

The set of probable initiating oncogenes in melanocytic tumors includes activating point mutations in *BRAF, NRAS, GNAQ, GNA11*, and activating fusions of *BRAF* and the receptor tyrosine kinases (RTKs) *ALK, ROS1, RET, MET*, and *NTRK1*. These mutations have been identified in benign and malignant melanocytic tumors in a mutually exclusive pattern, e.g. only one of these MAPK-activating mutations will be present. While *KIT* mutations do not co-occur with probable initiating oncogenes, they have not been identified in benign melanocytic tumors and it is unclear when activating *KIT* mutations arise during melanoma progression.

Initiating oncogenes may influence tumor phenotype. Different histopathologic subtypes of nevi demonstrate varying spectra of initiating mutations. Common acquired nevi harbor BRAF ^V600E^ mutations in ~85% of cases with activating *NRAS* mutations in 3–5% ^4^. The majority of blue nevi harbor activating mutations in one of two highly homologous members of the G-alpha Q family, *GNAQ* (65%) and *GNA11* (9%) ^5, 6^. Spitz nevi have the most diverse set of initiating mutations with activating *HRAS* mutations (22%) and activating fusions of *BRAF* (7%) and the RTKs *ALK* (12%), *MET* (2%), *NTRK1* (12%), *RET* (4%), and *ROS1* (25%) ^7– 10^.

Perhaps the phenotypic differences between common acquired nevi and blue nevi are due to their distinct initiating oncogenes. If so, the diversity of initiating oncogenes in Spitz nevi and tumors could explain the phenotypic variability and the diagnostic challenges of this class of tumors. Initial studies suggest that fusions of specific RTKs may result in specific histopathologic features. Specifically, Spitz tumors with ALK fusions commonly have distinctive vertically oriented plexiform nests of fusiform melanocytes ^11, 12^. Classification of Spitz tumors by the category of initiating oncogene may result in more refined histopathologic diagnostic criteria. One caveat is the diversity within a given class of fusion kinases. Structural rearrangements lead to oncogenic RTK fusion genes because the N-terminal fusion partner replaces the regulatory portion of the RTK. Without the regulatory domain, the kinase domain is constitutively active. Early findings indicate that activating fusions of the same RTK may be highly diverse in melanocytic tumors with a broad range of N-terminal partners fused to variable portions of the RTK ^8– 10^. The N-terminal partner influences expression, localization, and dimerization of the fusion kinase, all features expected to impact oncogenic signaling and thus potentially tumor phenotype.

## Progression events

The accumulation of oncogenic events in addition to an initiating event leads to melanoma. Owing to the high number of mutations observed in melanoma, distinguishing driver from passenger events is difficult and requires functional validation. Through large-scale sequencing studies, many progression events have been nominated in melanoma, but the functional consequences of most of them remain to be determined ^13– 15^. Understanding how combinations of oncogenic mutations interact and predict biologic behavior is an area of active investigation.

First identified in 2012 in familial and sporadic cutaneous melanoma, *TERT* promoter mutations result in a *de novo* E26 transformation-specific (ETS) factor binding site and increased TERT expression ^16– 18^. TERT is the enzymatic subunit of telomerase, and elevated telomerase activity prevents critical telomere shortening with cell division and bypasses replicative senescence. *TERT* promoter mutations are associated with worse prognosis in non-acral cutaneous melanoma and Spitzoid melanoma ^19, 20^.

A recent study of melanoma progression characterized various portions of melanocytic tumors that contained benign, intermediate, and malignant areas (melanoma arising within a nevus). *TERT* promoter mutations were identified in several “likely benign” intermediate melanocytic tumors and melanomas ^3^. The presence of *TERT* promoter mutations in combination with either *BRAF* or *NRAS* activating mutations in “likely benign” intermediate tumors suggests that these combinations of oncogenic mutations are not sufficient for malignant transformation. This finding demonstrates that an intermediate category of melanocytic neoplasia exists and corresponds with existing histopathologic classifications.

A recent study identified a novel mechanism that results in translation of the kinase portion of an RTK without its corresponding regulatory domain. A novel transcript of *ALK* transcribed from an alternative transcription initiation (ATI) site in intron 19 of the full-length isoform of *ALK* encodes the kinase domain of ALK without the extracellular or transmembrane regions. Present in ~3–11% of melanomas, ALK ^ATI^ is not associated with DNA sequence alterations of *ALK*. Rather, it appears that the expression of ALK ^ATI^ occurs due to epigenetic modification. *In vitro*, ALK ^ATI^ constitutively activates MAPK, AKT, and STAT3 signaling and is inhibited by small molecule ALK inhibitors. While the signaling output of ALK ^ATI ^is similar to that of ALK fusions, ALK ^ATI^ is seen in melanomas with and without activating *BRAF* and *NRAS* mutations, indicating that it is not an initiating event ^21^.

Biallelic *BAP1* loss can in many cases be distinctly identified by histopathologic examination. BAP1 is a histone deubiquitinase that functions as a tumor suppressor. It is recurrently inactivated in uveal melanoma ^22^. Germline loss-of-function variants increase the risk of melanoma, renal cell carcinoma, mesothelioma, and other cancers ^23, 24^. The distinctive cutaneous melanocytic tumors in patients with *BAP1* germline mutations are characterized by dermal epithelioid melanocytes with abundant eosinophilic cytoplasm and variably enlarged, pleomorphic, and eccentrically placed nuclei, often in a background of lymphocytic inflammation. These neoplasms harbor activating *BRAF* or *NRAS* mutations in addition to biallelic loss of *BAP1* and an adjacent common acquired nevus is often appreciated ^25, 26^. These findings are consistent with clonal expansion of a neoplastic melanocyte in a common acquired nevus (with *BRAF* or *NRAS* activating mutation) after biallelic loss of *BAP1*. Based on their cytomorphology, epithelioid tumors with *BAP1* loss were historically classified as atypical Spitz tumors or halo Spitz nevi, both considered to have negligible to low malignant potential.

Epithelioid tumors with *BAP1* loss (or Wiesner nevi) are distinct from other genetic categories of Spitz nevi in that three oncogenic mutations have occurred (activating *BRAF* or *NRAS* mutation and two hits to *BAP1*) in contrast to Spitz nevi with *HRAS* mutation or kinase fusions. Their characteristic cytomorphology is due to a progression event (loss of *BAP1*) rather than a direct effect of the initiating oncogene, as is hypothesized for other Spitz nevi. Thus, there is an argument to be made to cleave these tumors from the Spitz progression series and add them as a subtype of intermediate tumor on the *BRAF/NRAS* progression series.

Early observations indicate that *BAP1* loss in combination with *BRAF* or *NRAS* mutation gives rise to a low-risk melanocytic tumor (a topic worthy of further investigation). In contrast, BAP1 loss in combination with *GNAQ* or *GNA11* has not been identified in low-risk melanocytic tumors but occurs in uveal melanoma and melanoma arising in blue nevi (MABN) ^22, 27^. Loss of *BAP1* is associated with poor prognosis in uveal melanoma ^28^. Thus, the contribution of *BAP1* loss to malignant transformation in melanoma appears to differ depending on the initiating oncogene. Our models of melanoma progression will need to accommodate this complexity.

## Genomic reflections of aberrant cellular processes

Arm-level and whole chromosome gains and losses, as well as focal amplifications and deletions of the genome, are frequent in melanoma and uncommon in nevi. Copy number aberrations (CNAs), particularly when multiple, may reflect previous or ongoing genomic instability. Genomic instability can result from multiple disrupted biologic processes (oncogene-induced replicative stress, defective DNA damage response, or impaired cell cycle checkpoints).

The overrepresentation of specific copy number alterations in melanoma indicates selective advantage for specific CNAs (i.e. loss of *CDKN2A* or amplification of *CCND1*) and a role in tumor progression. Melanomas arising on chronically sun-damaged skin, non-chronically sun-damaged skin, acral glabrous skin, and mucosal epithelium have different patterns of CNAs, suggesting different causes of genomic instability and/or different pathways of genetic evolution ^29^. Not all types of melanoma demonstrate a high frequency of CNAs: for example, desmoplastic melanomas have few CNAs and a high number of single base substitutions ^30^.

While CNAs may reflect genomic instability, they can also result from stochastic events in the absence of a long-term cellular state of global genomic instability. These events include double-stranded DNA breaks and catastrophic events that lead to complex genomic rearrangements, such as chromothripsis ^31^. In benign or low-grade melanocytic tumors, such a chance event is thought to give rise to CNAs that lead to selective advantage and selection. Gain of chromosome 11p is often observed in *HRAS* mutant Spitz nevi ^7^. Monosomy 3 or focal loss including 3p21 is often observed in epithelioid tumors with biallelic loss of *BAP1* ^23, 25^. Identification of these isolated CNAs in the context of a tumor with the expected histopathologic characteristics does not lead to a diagnosis of melanoma. Copy number transitions within kinases may indicate a kinase fusion. Often times we observe probable “passenger” structural variants in the vicinity of kinase fusions (for example, the reciprocal fusion junction). The clinical significance of varying patterns of copy number alterations seen in association with kinase fusions remains to be determined.

## Molecular assessment for diagnosis

Assessment of copy number status has been used to supplement the histopathologic assessment of diagnostically challenging melanocytic tumors for over a decade. Array comparative genomic hybridization (aCGH) and fluorescence *in situ* hybridization (FISH) are in routine use by several diagnostic laboratories. One of the first FISH tests proposed for melanoma diagnosis employs four probes, assessing for gains of *CCND1* and absolute or relative gain of 6p or loss of 6q as compared to centromere 6 ^32, 33^. Additional FISH probes have been proposed for specific subtypes of melanocytic tumors (9p21 to assess for homozygous *CDKN2A* deletion in spitzoid tumors and 8q24 *MYC* gain to improve sensitivity in nevoid melanomas) ^34, 35^.

aCGH gives a broader assessment of copy number status but is less sensitive in the setting of low tumor purity and for subclonal CNAs and also requires more tissue than FISH. The patterns of CNAs are varied and the significance of a limited number of CNAs that are not common in melanoma remains to be determined. The copy number profile can provide clues to oncogenic alterations. For example, *KIT* amplification is often associated with *KIT* mutation, and copy number transitions in kinases with relative gain of the kinase portion of the gene may indicate an activating kinase fusion.

Initial studies highlight the promise of assessment of combinations of genetic alterations and expression profiles using multiplex analysis of DNA or RNA in the diagnosis of melanocytic neoplasia ^3, 36^. Additional studies with clinical follow-up and stratification by histopathologic and genetic subtype will inform how best to integrate these complex tests into current clinical practice.

## Molecular assessment for treatment selection

Currently, the two major approaches to the treatment of metastatic melanoma are immunotherapy and molecularly targeted therapies, and there are studies underway to evaluate combination regimens. The checkpoint inhibitors ipilimumab (anti CTLA-4 antibody), nivolumab, and pembrolizumab (anti PD-1 antibodies) result in objective responses in 10–40% of patients and an overall survival benefit ^37– 40^. PD-L1 expression correlates with response to anti PD-1 antibodies, and the combination of nivolumab and ipilimumab improves response rates in PD-L1-negative tumors. As the side effect profile of checkpoint inhibitors is not insignificant, work is currently ongoing to identify which patients will benefit from these treatments. In non-small-cell lung cancer, a higher mutation burden (likely a proxy for increased neoantigens) is associated with improved response to immunotherapy ^41^. Estimation of mutation burden, neoantigen expression, or expression profile may refine therapy selection for metastatic melanoma in the near future ^42^.

Targeted therapy of BRAF ^V600E^ mutant melanoma with inhibitors of mutant *BRAF* is currently part of the standard of care. Combination with MEK inhibitors improves outcomes ^43, 44^. Approximately 50% of metastatic melanomas harbor a *BRAF* mutation, ~25% harbor an activating *NRAS* mutation, and 3–5% harbor an activating *KIT* mutation. Inhibitors of NRAS are currently unavailable, but initial clinical trials of MEK inhibitors in NRAS mutant melanomas show some efficacy ^45^. Dramatic responses to KIT inhibitors such as imatinib and nilotinib have been observed in patients with *KIT* mutant melanoma ^46– 50^.

In a minority of cutaneous melanoma patients, an activating mutation in *BRAF*, *NRAS*, or *KIT* is not identified. In these patients, testing for a kinase fusion may yield a potential therapeutic target. In case reports of patients with *BRAF* fusion melanoma, responses to sorafenib and trametinib were observed ^8, 51, 52^. Treatment of other solid tumors with RTK fusions similar to those observed in melanoma provides clinical benefit as exemplified by ALK inhibition in lung cancer with ALK fusions ^53, 54^. Clinical studies are needed to assess the efficacy of kinase inhibitors for kinase fusion melanoma.

There are an increasing number of diagnostic modalities available for the detection of actionable and potentially actionable genetic alterations. Considerations for selecting specific assays include cost, turn-around time, comprehensiveness for actionable alterations (a moving target), and specimen requirements. For point mutations, immunohistochemistry (VE1 for BRAF ^V600E^ and SP174 for NRAS ^Q61R^) and allele-specific real-time polymerase chain reaction (RT-PCR) assays (cobas® 4800 BRAF V600) provide quick, highly sensitive, and easy-to-interpret assessment for a narrow spectrum of mutations ^55– 57^. Sanger sequencing has been traditionally used for the detection of hotspot mutations in oncogenes and can detect mutations within the assayed region (i.e. *BRAF* exon 15). One limitation of Sanger sequencing is a limit of detection of ~10–20% minor allele frequency (corresponding to 20–40% tumor fraction in a heterozygous sample), resulting in decreased sensitivity for samples with low tumor fraction. Next-generation multiplex sequencing is being increasingly adopted as a way to perform multiplex testing of oncogenes with a lower limit of detection owing to the ability to sequence individual DNA molecules. Next-generation sequencing (NGS) cancer testing platforms typically assess a panel of oncogenes that are of interest in many types of cancer. By broadening the regions of the genome assayed, these panels may detect alterations that are actionable in other cancer types and rare in melanoma. These assays can also detect CNAs.

One can take advantage of the mutual exclusivity of actionable alterations and their prevalence in melanoma to perform stratified testing of a tumor sample. Given the high rate of BRAF V600 mutations, V600E-specific testing (immunohistochemistry or real-time based assay) or *BRAF* exon 15 testing (Sanger) followed by a test for a broader panel of oncogenes (including *NRAS* and *KIT*) if a *BRAF* mutation is not detected could optimize cost and turn-around time for melanoma patients, depending on testing strategies employed.

Identification of kinase fusions requires different approaches than the detection of oncogenic hotspot mutations, as the genomic breakpoints usually occur in intronic regions that span a much larger portion of the genome than hotspot coding mutations. Detection of fusion transcripts by RT-PCR is highly sensitive (i.e. BCR-ABL in chronic myelogenous leukemia), but RT-PCR is not practical for detecting the broad spectrum of kinase fusions that occur in melanoma. Immunohistochemistry to assess the expression of the kinase domain of ALK, ROS1, NTRK1, and MET appear to be highly sensitive for detecting fusion kinases but with varying specificity. The lack of specificity can be due to basal expression of the kinase in melanocytes (NTRK1 and MET) and alterative oncogenic mechanisms that lead to expression of the kinase domain (ALK ^ATI^). Hybrid-capture-based NGS DNA assays can detect structural rearrangements that lead to oncogenic fusions by sequencing the introns in which the breakpoints occur and can be multiplexed with detection of other melanoma oncogenes, but this method has limited sensitivity due to repetitive regions within introns and the technical difficulty of identifying structural rearrangements from short-read sequencing. Multiplex RNA-based methods are more sensitive. FISH break-apart probes are also available for fusion detection.

## Future directions

The rapid pace of technologic development has led to a remarkable expansion of our understanding of the genetic progression of cancer and melanoma. Translation of these findings into the clinic is exceeding at a rapid pace. As always, we are treating patients with the best information we have on hand while pushing for additional studies to support our current best practices in diagnosis and treatment. Refining our understanding and models of genetic progression will help us develop the best clinical and biologic hypotheses to direct future investigation.
